# Effect of cement distribution type on clinical outcome after percutaneous vertebroplasty for osteoporotic vertebral compression fractures in the aging population

**DOI:** 10.3389/fsurg.2022.975832

**Published:** 2022-08-08

**Authors:** Chengqiang Zhou, Yifeng Liao, Shaolong Huang, Hua Li, Ziqiang Zhu, Li Zheng, Bin Wang, Yunqing Wang

**Affiliations:** ^1^ Department of Orthopedics, The Second Affiliated Hospital of Xuzhou Medical University, Xuzhou, China; ^2^ Graduate School of Xuzhou Medical University, Xuzhou, China

**Keywords:** percutaneous vertebroplasty, osteoporotic vertebral compression fracture, bone cement distribution, osteoporosis, clinical efficacy

## Abstract

**Objective:**

The study aimed to investigate the effect of the type of bone cement distribution on clinical outcomes following percutaneous vertebroplasty (PVP) for osteoporotic vertebral compression fractures (OVCF) in the elderly.

**Methods:**

Retrospective analysis of 160 patients diagnosed with OVCF who underwent PVP treatment from March 2018 to December 2020. Based on the kind of postoperative bone cement distribution, bone cement was classified as types I, II, III, IV, and V. Visual Analog Scale (VAS), Oswestry Disability Index (ODI), Cobb angle, anterior vertebral height ratio, refracture rate of injured vertebrae, and incidence of adjacent vertebral fractures were compared for the five types before and after three days, and one year of operation.

**Results:**

VAS and ODI at three days and one year postoperative were significantly lower than those preoperative (*P* < 0.05) for all five distribution types. VAS and ODI for types I, II, and III were lower at one year postoperatively than for types IV and V (*P* < 0.05). There was no significant difference in Cobb angle and anterior vertebral body height ratio between preoperative and three days postoperative groups (*P* < 0.05); however, there were significant differences between three days and one-year postoperative and preoperative groups (*P* < 0.05). Following one year of surgery, the Cobb angle and the anterior vertebral height ratio of types IV and V were significantly different from those of types I, II, and III (*P* < 0.05), and there was a statistically significant difference between types IV and V (*P* < 0.05). In terms of the incidence of injured vertebral refractures and adjacent vertebral fractures, the evenly distributed types I, II, and III were significantly lower than the unevenly distributed types IV and V, and the incidence of type V was higher (*P* < 0.05).

**Conclusions:**

The clinical efficacy of cement distribution following PVP of types I, II, and III is better than that of types IV and V, which can better relieve pain with long-lasting efficacy and minimize the occurrence of refractures of injured vertebrae and adjacent vertebral body fractures.

## Introduction

The incidence of osteoporotic vertebral compression fractures (OVCF) has increased yearly as the population ages (1). Percutaneous vertebroplasty (PVP) or percutaneous kyphoplasty (PKP) is an internationally recognized minimally invasive procedure for treating OVCF with low trauma and rapid recovery. It has been widely used in treating OVCF in the thoracolumbar spine with proven efficacy, becoming the gold standard for treating OVCF patients (2, 3). The mechanism of vertebroplasty is to fix microfractures and enhance the stability of the vertebral body by injecting bone cement into the vertebral body (4). Thus, the distribution of bone cement in the vertebral body is linked to clinical efficacy (5, 6). We investigated the correlation between the distribution type and clinical outcome by retrospectively analyzing the distribution type of postoperative bone cement in 160 patients with OVCF treated with PVP from March 2018 to December 2020, as reported below.

## Materials and methods

### Information

#### General information

Inclusion criteria: (1) osteoporotic patients, T-score of bone mineral density (BMD) ≤ −2.5; (2) single-segment osteoporotic vertebral compression fracture of the thoracolumbar spine; (3) age 60–85 years; (4) compression ratio of the injured spine ≤ 1/3; (5) MRI examination of the vertebral body T2W1 showed high signal, fat suppression sequence imaging with the presence of edema signal, and confirmed the diagnosis of OVCF; (6) imaging examination of the posterior wall and pedicle of the injured spine was intact, and there was no compression of the spinal canal. (7) The surgical method was PVP, and there was no postoperative cement leakage and spinal nerve injury; (8) patients with complete clinical, imaging, and follow-up data, or patients who could be followed up retrospectively; (9) the distribution of the bone cement in the lateral position was all located in the anterior 2/3 of the vertebral body and as close as possible to the upper endplates. Exclusion criteria were as follows: (1) patients unable to tolerate surgery prone; (2) patients with pathological fractures caused by tumor or infection; (3) patients with severe medical diseases.

#### Classification method

**Figure 1:**
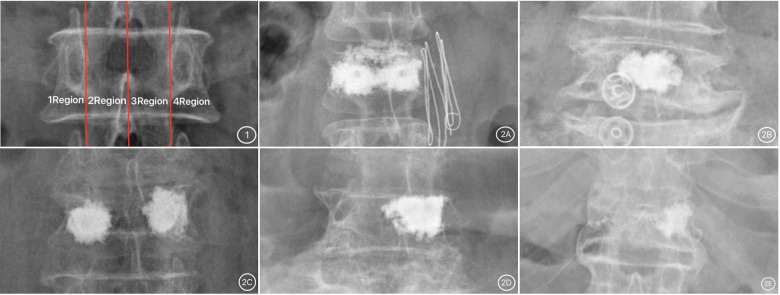
(1) Vertebral body division picture; (2) bone cement distribution pictures. (**2A**) Type I; (**2B**) Type II; (**2C**) Type III; (**2D**) Type IV; (**2E**) Type V.

Based on the frontal X-ray of the vertebral body, three vertical lines were drawn in the middle of the central spinous process and the inner edge of the pedicles on both sides; as a result, the vertebral body was classified into 1 to 4 regions. The distribution pattern of bone cement was divided into five types based on the distribution location of bone cement in the vertebral body, the first of which was the type I, where most of the bone cement was continuously and evenly distributed in the vertebral body (regions 1–4); type II, where most of the bone cement was distributed in the central part of the vertebral body (regions 2 and 3); type III, with most of the bone cement, distributed on both sides of the vertebral body (regions 1 and 4); type IV, in which most of the bone cement was concentrated on one side and in the center of the vertebral body (regions 1 and 2, or regions 3 and 4); type V, most cement was concentrated on one side of the vertebral body (region 1 or 4). Note on classification: “Most of the cement” referred to the main body of the cement, not “all of the cement” (7) (Figure 1).

#### Grouping data

A total of 160 patients with OVCF fractures were included. The study comprised 52 males and 108 females aged 60–83 years (mean: 68.91 ± 8.19 years), with a body mass index (BMI) of 14.07–32.09 kg/m^2^ (mean: 22.55 ± 3.23 kg/m^2^), BMD of −4.12–2.48 (mean: −3.22 ± 0.31), operating time of 32–58 min (mean: 44.89 ± 5.40 min), and follow-up time of 12–27 months (mean: 18.02 ± 4.02 months). Lesioned vertebral body sites: T6 in four cases, T7 in six cases, T8 in five cases, T9 in seven cases, T10 in 13 cases, T11 in 13 cases, T12 in 17 cases, L1 in 24 cases, L2 in 30 cases, L3 in 26 cases, L4 in 10 cases, and L5 in five cases. Bone cement distribution type was as follows: Type I: 37 cases, 12 males and 25 females; age 60–80 years, mean age 70.5 years. Type II: 31 cases, 10 males and 21 females; age 60–81 years, mean age 71.5 years. Type III: 35 cases, 13 males and 22 females; age 62–83 years, mean age 72.5 years. Type IV: 29 cases, nine males and 20 females; age 61–83 years, mean age 70.6 years. Type V: 28 cases, eight males and 20 females; age 61–82 years, mean age 69.3 years. There was no statistically significant difference between the data of gender, age, BMI, BMD, operation time, and follow-up time of patients of each type (*P* > 0.05), which were comparable, as shown in Table 1.

**Table 1:** General data.

Distribution pattern	*n*	Gender	Age (years)	BMI (kg/m^2^)	BMD (T score)	Operation time (mins)	Follow-up time (months)
I	37	12/25	68.19 ± 7.84	22.09 ± 2.32	−3.12 ± 0.35	44.59 ± 5.73	18.89 ± 5.12
II	31	10/21	69.74 ± 8.50	22.73 ± 3.75	−3.17 ± 0.19	46.68 ± 6.18	17.39 ± 4.95
III	35	13/22	69.43 ± 7.06	23.18 ± 3.96	−3.26 ± 0.35	45.91 ± 5.04	18.03 ± 4.80
IV	29	9/20	70.38 ± 7.49	22.93 ± 2.90	−3.23 ± 0.33	44.72 ± 4.86	18.07 ± 5.08
V	28	8/20	69.96 ± 6.34	23.01 ± 3.69	−3.30 ± 0.29	45.18 ± 6.10	17.21 ± 4.82
t/x^2^		0.570	0.417	0.556	1.763	0.785	0.593
*P*		0.966	0.796	0.695	0.139	0.536	0.668

### Methods

#### Surgical methods

The surgeries were performed by clinically experienced surgeons who strictly followed the same operation protocol. The patient was placed in a prone position with hands raised and shoulder and pelvic cushions. The surgical bed was reversed in a V-shape to allow posterior spine extension for repositioning. G-arm fluoroscopy was used to locate and observe the repositioning of the compressed vertebrae. After reaching the requirements, the procedure was performed under local anesthesia with routine disinfection and towel laying. The puncture and surgical instruments of matching diameters were selected according to the puncture site of the thoracolumbar spine. The 3.0 mm diameter puncture needle was used for the lumbar and lower thoracic vertebrae. The puncture was performed under G-arm fluoroscopic monitoring with a bilateral pedicle approach. After a successful puncture, the 4.2 mm working sleeve was replaced, inserted into the vertebral body, and drilled to the anterior middle and lower 2/3 of the vertebral body. A bone cement push rod was inserted, and bone cement from the mid to late stages of the draw was slowly and repeatedly pushed into the vertebral body under X-ray fluoroscopy, no more than 0.3 ml at a time, and observed for leakage of bone cement. Antibiotics were used for 1–3 days following surgery, and after 1–3 days of bed rest, the waist circumference could be assisted to get out of bed, and anti-osteoporosis treatment was standardized after discharge.

### Main observation indicators

The basic data of each type of patient were recorded and compared, including pain VAS, ODI, Cobb angle, anterior vertebral height ratio, and the incidence of refracture of the injured vertebra and adjacent vertebral fracture; one day before surgery, three days and one year after surgery. ODI was based on the Oswestry dysfunction index scale excluding sexual life, and the total value of the actual score/45 was used. The Cobb angle was measured by drawing a horizontal line at the upper edge of one cone on the injured vertebra and a horizontal line at the lower edge of the lower cone on the damaged vertebra; the angle of intersection of the two lines was the Cobb angle. The anterior vertebral height ratio is measured at the most significant point of vertebral compression/the average of the measurements of the same part of the adjacent upper and lower vertebrae × 100%.

### Statistical methods

Statistical analysis was performed on all data in this study using SPSS 26.0. The measurement data were tested for normality and conformed to normal distribution. Multiple group comparisons were conducted using one-way ANOVA, and pairwise comparisons were conducted using the SNK-q test, expressed as $\bar{\chi }\pm s$; count data were tested by *χ*^2^ test or Fisher's exact probability method, expressed as a rate (%). A difference in statistical significance was indicated by *P* < 0.05.

## Results

### Comparison of basic data

There was no significant difference between all categories of patients (*P* > 0.05) when basic characteristics such as gender, age, BMI, BMD, operation time, and follow-up time were compared (Table 1).

### Comparison of VAS and ODI between groups

There was no significant difference in VAS and ODI between groups before and three days after surgery (*P* > 0.05). Still, VAS and ODI were significantly reduced (*P* < 0.05) at three days and one year after surgery than before. However, VAS and ODI of types IV and V at one year postoperatively were significantly higher than those of types I, II, and III (*P* < 0.05), as shown in Table 2.

**Table 2:** Comparison of VAS and ODI between groups.

Typing	*n*	VAS			ODI		
		preoperative	3 d postoperative	1 year postoperative	preoperative	3 d postoperative	1 year postoperative
I	37	7.32 ± 0.94	2.35 ± 0.54^a^	1.41 ± 0.50^a^	37.70 ± 2.54	20.19 ± 2.84^a^	12.97 ± 3.03^a^
II	31	7.55 ± 0.89	2.42 ± 0.67^a^	1.45 ± 0.57^a^	37.29 ± 2.37	20.68 ± 2.53^a^	11.94 ± 3.13^a^
III	35	7.14 ± 0.55	2.60 ± 0.69^a^	1.51 ± 0.51^a^	37.57 ± 2.40	19.94 ± 2.67^a^	13.40 ± 2.79^a^
IV	29	7.34 ± 1.14	2.66 ± 0.55^a^	1.97 ± 0.63^a^^,^^b^	37.28 ± 2.78	21.10 ± 3.24^a^	16.72 ± 3.23^a^^,^^b^
V	28	7.21 ± 0.57	2.54 ± 0.69^a^	2.07 ± 0.66^a^^,^^b^	38.14 ± 3.11	20.43 ± 2.87^a^	17.89 ± 3.05^a^^,^^b^
t		1.057	1.313	9.217	0.539	0.795	21.598
*P*		0.380	0.268	<0.001	0.708	0.530	<0.001

Note: Compared with preoperative.

^a^ *P* < 0.05; compared with type I, II, and III.

^b^ *P* < 0.05.

### Comparison of Cobb angle and anterior vertebral height ratio between groups

There was no significant difference in Cobb angle and anterior vertebral body height ratio between the preoperative and three days postoperative groups (*P* > 0.05); however, there were significant differences between three days and one-year postoperative as compared to preoperative (*P* < 0.05). After one year postoperatively, there were statistically significant variations in Cobb angle and anterior vertebral body height ratio between types IV, V, and types I, II, and III (*P* < 0.05), and there was a statistically significant difference (*P* < 0.05) between types V and IV (Table 3).

**Table 3:** Comparison of Cobb angle and anterior vertebral body height ratio between groups.

Typing	*n*	Cobb angle			anterior vertebral height ratio		
		preoperative	3 d postoperative	1 year postoperative	preoperative	3 d postoperative	1 year postoperative
I	37	14.45 ± 3.44	6.80 ± 1.92^a^	6.25 ± 2.52^a,^^c^	61.51 ± 10.63	83.94 ± 5.62^a^	82.91 ± 5.51^a,^^c^
II	31	14.30 ± 4.65	7.60 ± 2.31^a^	7.56 ± 2.13^a,^^c^	59.46 ± 11.10	82.34 ± 6.01^a^	82.69 ± 5.99^a,^^c^
III	35	16.01 ± 2.42	7.59 ± 2.38^a^	8.03 ± 3.02^a.^^c^	59.18 ± 9.77	82.92 ± 5.94^a^	82.18 ± 6.00^a,^^c^
IV	29	15.81 ± 2.72	7.81 ± 3.12^a^	10.31 ± 2.94^a,^^b,^^c^	59.83 ± 10.32	80.98 ± 5.42^a^	77.83 ± 5.34^a,^^b,^^c^
V	28	14.90 ± 2.62	8.15 ± 3.22^a^	12.88 ± 2.75^a,^^b^	60.01 ± 11.39	80.27 ± 5.91^a^	73.11 ± 5.90^a,^^b^
*t*		1.866	1.229	29.029	0.261	2.076	16.367
*P*		0.119	0.301	<0.001	0.903	0.087	<0.001

Note: Compared with preoperative.

^a^ *P* < 0.05; compared with type I, II, and III.

^b^ *P* < 0.05; compared with type V.

^c^ *P* < 0.05.

### Comparison of refractures of injured vertebrae and adjacent vertebral fractures between groups

**Figure 2:**
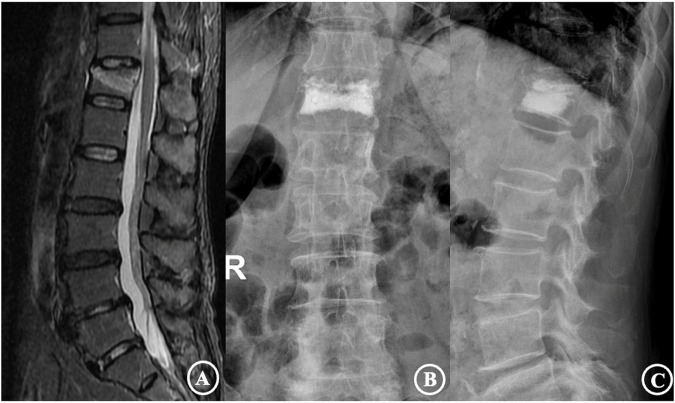
The patient was a 72-year-old female diagnosed with an osteoporotic vertebral compression fracture of T12. (**A**) is a preoperative MRI film suggesting a fresh vertebral compression fracture of the T12 vertebra; (**B**,**C**) are frontal and lateral X-rays after percutaneous vertebroplasty, showing a type I cement distribution and good cement dispersion.

**Figure 3:**
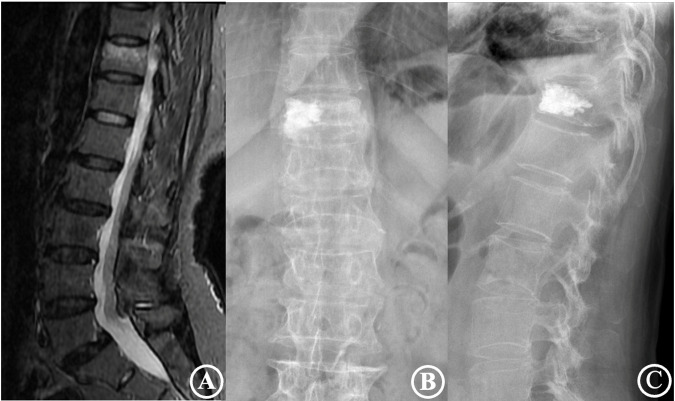
The patient was a 75-year-old male diagnosed with an osteoporotic vertebral compression fracture of T11. (**A**) is a preoperative MRI film suggesting a fresh vertebral compression fracture of the T11 vertebra; (**B**,**C**) are frontal and lateral X-rays after percutaneous vertebroplasty, showing a type IV cement distribution and poor cement dispersion.

The distribution of 160 reinforced vertebrae in T6-L5, the occurrence of injured vertebrae refracture, and adjacent vertebrae fracture are shown in Table 4. The results revealed that the incidence of refractures of injured vertebrae and adjacent vertebral body fractures in patients with types IV and V was significantly higher than that of types I, II, and III and were higher in type V than type IV. The differences were statistically significant (*P* < 0.05). Typical cases are displayed in Figures 2, 3.

**Table 4:** Comparison of refractures of injured vertebrae and adjacent vertebral fractures between groups.

	*n*	Positioning of injured vertebrae			Refracture of injured vertebrae			Fracture of adjacent vertebrae		
		>T10	T11–L2	L3–L5	>T10	T11–L2	L3–L5	>T10	T11–L2	L3–L5
I	37	7	18	12	0	0	0	0	1	0
II	31	4	19	8	0	0	0	0	1	0
III	35	7	18	10	0	1	0	0	3	0
IV	29	8	15	6	1	4	2	1	5	3
V	28	9	14	5	3	8	3	4	9	3

## Discussion

Studies have shown that PVP/PKP are superior to non-surgical treatment to relieve acute, subacute, and chronic osteoporotic vertebral compression fracture pain (8, 9). Therefore, vertebroplasty has become the preferred treatment strategy for OVCF. For OVCF patients with persistent severe pain, vertebroplasty intervention within two months is more effective (10, 11). However, factors such as the expertise and technical proficiency of the operating surgeon during vertebroplasty, as well as the duration of vertebral injury, cement viscosity, cement volume, and degree of osteoporosis, can all impact the therapeutic effect of the procedure. According to literature (12), insufficient intravertebral filling with bone cement is the primary reason for poor pain relief after vertebroplasty. Hence, Cement distribution in the vertebra during vertebroplasty is closely linked to pain relief and durability of curative effect after surgery. This is because the distribution of bone cement affects the strength and biomechanical stability of the strengthened vertebral body (13). There is currently no simple and practical approach for evaluating the distribution of bone cement in clinical practice. In this study, the vertebral body was divided into four regions based on the central vertical line and the vertical line of the inner edge of the bilateral pedicles on the postoperative frontal X-ray film, and it was classified into five groups, I to V, based on the different types of postoperative bone cement dispersion. This typing approach is simple, easy to use, highly reliable, reproducible, more accurate, refined, and standardized than diffusion shape typing alone. Moreover, it can well meet the needs of clinical evaluation. If there is bone cement leakage in this study, even if it does not cause clinical symptoms, it should be excluded to avoid the impact of cement leakage on adjacent vertebral refracture. For this reason, typing on lateral films was not performed in this study.

In the current study, the postoperative anterior X-ray films were examined, and it was observed that the distribution of bone cement was different, and the postoperative effects were not the same. Therefore, it was speculated that the distribution of bone cement influenced the surgical effect. Bone cement types I, II, III (uniform distribution), and IV, V (non-uniform distribution) significantly reduced the postoperative VAS and ODI of OVCF patients, suggesting that the injection of bone cement into the diseased vertebrae stabilized microfractures and damaged nerve endings in the vertebral body. Moreover, it was evident that the efficacy of this technique was significant, and it could effectively relieve pain and offer clinical effectiveness. However, the follow-up found that VAS and ODI of patients with bone cement diffusion for types I to III was lower than those of type IV to V patients one year after the operation, indicating that the asymmetric distribution of bone cement will affect the long-term efficacy of vertebroplasty. The reason might be that there was no bone cement filling on the contralateral side of the vertebral bodies of type IV and V distribution, leading to failure to stabilize local microfractures and destroy local nerve endings and the lack of pain symptoms relief in the unfilled test due to biased load shifting inside the vertebral body. There is also a correlation between the efficacy of vertebroplasty and the vertebral height and Cobb angle improvements. It has been reported that vertebroplasty can partially restore the height of the injured vertebra and rectify spinal deformity (14–16). This study's findings demonstrated that PVP reduces the Cobb angle and restores the anterior vertebral height ratio without considering the distribution of bone cement. If the bone cement is distributed in type I-III within the vertebral body, the vertebral body height can be better maintained, and the risk of postoperative local kyphosis and vertebral body height loss can be reduced. This results from the dispersion of type I–III bone cement, which enables the bone cement and cancellous bone to be more closely linked, thereby increasing the strength and stiffness of the vertebrae and decreasing the likelihood of vertebral height loss and kyphosis deformity following PVP (17). Types IV and V cement cannot adequately fill the fractured vertebral body, resulting in decreased strength and stiffness of the vertebrae and, as a result, are unable to provide effective support, which causes an increase in the Cobb angle and a decrease in the vertebral height ratio (18).

Through retrospective analysis of case data, we believe that the different types of postoperative bone cement dispersion are connected to the segment of the fractured vertebral body, the puncture angle, the degree of osteoporosis, and the degree of compression of the injured vertebrae. Due to the different segments of the injured vertebrae, the closer to the upper and middle thoracic vertebrae, the smaller the volume of the vertebral body, especially the high thoracic vertebrae, which are difficult to puncture. Because the unilateral parapedicular technique is often employed for a puncture, the postoperative cement distribution is higher in type IV or V. Therefore, for patients with thoracic OVCF, bilateral pedicle puncture promotes good bone cement diffusion, which is significant for increasing postoperative clinical efficacy. When the bilateral puncture is performed, because there are bilateral channels, the bone cement can be injected at a later stage of lasing. Simultaneously, bilateral injection of bone cement makes the bone cement more uniformly distributed in the vertebral body. Therefore, bilateral punctures should be performed whenever possible; there is no significant difference in trauma and operative time in bilateral and unilateral punctures (19). If the patient cannot tolerate the procedure physically for an extended period, the unilateral puncture is required to reduce operative time by increasing the puncture angle during the procedure, while the cement injection point should be close to the midline. The lower thoracic and lumbar vertebrae are often treated using a puncture approach with a relatively large volumes of cement injection, which is common for types I to III. In patients with types IV and V, the puncture angle should be increased intraoperatively, and bilateral punctures should be used as often as possible to compensate for poor diffusion. Also, in order to exclude the impact of the involved segments of different patients on the results of bone cement distribution after PVP, we observed 84 T11 to L2 vertebrae in this study individually. We found that the clinical efficacy of type I, II, and III bone cement distribution in T11 to L2 vertebrae remained superior to type IV and V.

This study demonstrated that maintaining the intraoperative bone cement dispersion to types I-III greatly reduces the incidence of postoperative refracture of adjacent vertebrae. Chevalier et al. (20) concluded that adequate dispersion of bone cement within the upper and lower endplates of the vertebral body reduced the incidence of postoperative vertebral body recollapse or fracture. Bone cement injection into the vertebral body shows different types and divisions. Type I bone cement diffuses to regions 1–4, where the injured vertebra has the best strength recovery, and the force balance between the injured vertebra and the adjacent vertebral bodies is the most stable. In types II and III, the bone cement is distributed in at least two parts of the vertebral body. The mechanics of the injured vertebra and the adjacent vertebral body remain balanced. Types IV and V (uneven distribution of bone cement), in which the majority of the bone cement is distributed in the pedicle area and a portion of the central area on one side of the vertebral body (type IV) or the main body of bone cement is distributed only on one side of the vertebral body (type V), the injured vertebra is in a state of imbalance with the adjacent vertebral body, which may lead to adjacent vertebral body fractures and refractures. In this study, one case of adjacent vertebral fracture occurred in type I, one in type II, three in type III, nine in type IV, and 16 in type V. Refractures after PVP mainly occurred in type IV and V patients. One probable explanation is that when the bone cement becomes IV and V type (unilateral distribution), the vertical compression force of the whole vertebral body shifts to the other side, increasing the vertical stress of the adjacent vertebral body. The vertebral body, however, is too stiff after being cemented, and the stresses are unevenly distributed, which will be transmitted to the intervertebral disc and the adjacent vertebral body, thus leading to fracture of the adjacent vertebral body and refracture of the injured vertebra. As a result, proper bone cement distribution is critical to reducing the risk of adjacent vertebral fractures. Liang et al. (21) used finite element analysis and found that the maximum von Mises stress in cancellous bone significantly increased in poorly cemented vertebrae compared to adequately cemented vertebrae, making them more prone to recollapse. Chen et al. (22) reported that the unilateral bone cement distribution made the stiffness of both sides of the vertebral body significantly different, resulting in unbalanced stress on both sides. Therefore, the bilateral symmetry should be maintained as much as possible during the injection of bone cement, as the asymmetric distribution will cause unbalanced stress transfer in the injured vertebra. Therefore, the type I dispersion is probably the ideal type. Types II and III dispersion can still achieve good clinical efficacy when type I dispersion cannot be satisfied.

In this study, in order to exclude the effect of different distribution of bone cement in the lateral position, we restricted the distribution of bone cement in the lateral position at the time of inclusion criteria. All included patients required that the distribution of bone cement in the lateral position should be located in the anterior 2/3 of the vertebral body and as close as possible to the upper endplates.

In clinical work, we should pay attention to the following points to achieve type I or symmetrical distribution as much as possible: (1) preoperative precise positioning and fluoroscopy should ensure the accurate location of the injured vertebral body and the needle insertion point; (2) when the puncture needle enters the pedicle, the puncture site should be positioned in the center lateral region of the articular process. Before the operation, a 2.0 thin guiding needle can be inserted into the pedicle, dependent on the position of the fracture target. According to the deviation of the guide needle from the fracture target, the puncture needle can be adjusted by altering the stress. (3) When puncturing the upper and middle thoracic vertebrae, the puncture angle should be inclined toward the cephalad, the abduction angle should be adequately increased, and bilateral puncture should be selected as much as possible. Under X-ray fluoroscopy, the bone cement should be filled sequentially through the working sleeve from vertebral body regions 2 and 3 to 1 and 4, and the direction of the puncture needle, as well as the position and amount of bone cement filling, should be moderately adjusted based on intraoperative fluoroscopic data and the direction of bone cement dispersion. (4) The injection timing should be determined according to the preoperative bone density and imaging, and if the osteoporosis is severe, the injection should be performed during the bone cement toothpaste period. If it is not severe, it can be administered during the drawing period to ensure adequate dispersion of bone cement; (5) If there is intraoperative bone cement leakage, the bone cement injection point should be stopped or adjusted in time.

The current study has certain limitations. First, this is a retrospective study, not a prospective, large-sample, multicenter study, and there is no empirical evidence for relevant *in vitro* biomechanical studies. Second, to observe the distribution of bone cement in the current investigation, the typing method was performed on two-dimensional X-ray anterior radiographs rather than three-dimensional stereoscopic images. Finally, there are many methods to define bone cement distribution, and further in-depth research is required for the distribution method used in this study. A new instrument is expected to improve the distribution of types I, II, and III bone cement. It is also believed that a new instrument can better achieve the distribution of types I, II, and III bone cements.

In conclusion, the evaluation method of bone cement distribution used in this study has the characteristics of easy operation, high reliability and repeatability, apparent differentiation of curative effect, accurate complications prediction, and certain clinical applicability. Simultaneously, bone cement type I distribution may provide the best clinical efficacy. If type I distribution cannot be attained, types II and III are acceptable suboptimal states.

## Data Availability

The raw data supporting the conclusions of this article will be made available by the authors, without undue reservation.
